# The Year’s Work

**Published:** 1887-12

**Authors:** 


### HOMŒOPATHIC PHYSICIAN,

A MONTHLY JOURNAL OF MEDICAL SCIENCE.

"If our school ever gives up the strict inductive method of Hahnemann, we are lost, and deserve only to be mentioned as a caricature in the history of medicine."—CONSTANTINE HERING.

Vol. VII. DECEMBER, 1887. No. 12.

### THE YEAR'S WORK.

It is with more than usual gratification that we close our year's work, and this gratification is on account of the very excellent journal our contributors have given us.

During the past year an unusually instructive and complete journal has been published, and the credit is almost entirely due to the zeal and energy of our able contributors. Should any one think we speak too boastfully, let him carefully examine the twelve numbers issued. By so doing the skeptic will be more than convinced—he will be astonished—for papers will be found on all subjects of interest to practitioners, written by leading homœopathists of this country and Europe.

It has been the aim of the editors to make THE HOMŒOPATHIC PHYSICIAN a teacher of pure Homœopathy, and more especially of the materia medica. In this particular we have always excelled all other journals, and we expect to continue to do so. The homœopathic materia medica is the basis of homœopathic practice. Without a pure materia medica there can be no homœopathic practice. Therefore the purity of the materia medica is simply a question of life or death to all true homœopaths.

While the large majority of journals are engaged in deriding, corrupting, or destroying the materia medica, we are trying to save it, to purify it, and to strengthen it. In pursuance of this purpose, articles of all kinds on the materia medica have been published (Lectures, "Notes," etc.). We have now undertaken to publish a complete rendering of the "characteristics." The first part of these "Notes" is given in this issue. Much labor has been spent on these "Notes," and it is hoped they will be of great use to physicians.

There is another very important point which calls for most careful consideration, and this is the purity of our drug preparations. Often do we hear it said that drugs do not act as well as they used to do. Carelessness seems to be creeping into our pharmacies. Let those interested in the manufacture of homœopathic medicines take warning. Pure medicines and a pure materia medica are absolutely and vitally essential to the success of the physician. To these subjects we shall continue to devote our attention.

